# Demography of the Gambian Epauletted Fruit Bat (*Epomophorus gambianus*) in Ghana

**DOI:** 10.1093/jmammal/gyae096

**Published:** 2024-09-05

**Authors:** Kofi Amponsah-Mensah, Michael A Hudson, Andrew A Cunningham, James L N Wood, Yaa Ntiamoa-Baidu

**Affiliations:** Centre for Biodiversity Conservation Research, Ebenezer Laing Road, GA 490-3153, University of Ghana, Legon, P.O. Box LG 67, Accra, Ghana; Durrell Wildlife Conservation Trust, Les Augres Manor, La Profonde Rue, Trinity JE3 5BP, Jersey; Institute of Zoology, Zoological Society of London, Regent’s Park, London NW1 4RY, United Kingdom; Disease Dynamics Unit, University of Cambridge, Madingley Road, Cambridge CB3 OES, United Kingdom; Centre for Biodiversity Conservation Research, Ebenezer Laing Road, GA 490-3153, University of Ghana, Legon, P.O. Box LG 67, Accra, Ghana

**Keywords:** birth rates, capture–mark–recapture, fruit bat, radiotelemetry, reproductive chronology, sex ratio, survival

## Abstract

We provide the first estimates of survival and reproductive rates for a population of the Gambian Epauletted Fruit Bat *Epomophorus gambianus* in Ghana. We focused on a large colony of ca. 5,000 bats over 3 years to estimate population parameters including population size, birth rates, survival, and sex ratios for this species. Reproduction chronology was confirmed as seasonal bimodal polyestry, with births occurring in March/April and August/September each year. The estimated birth rate was 0.89 (95% CI = 0.85 to 0.92) per reproductive season. The overall sex ratio (female to male ratio) of the study population was male-dominated (0.69, 95% CI = 0.64 to 0.75), but female-biased for adults (62% female, χ^2^_1_ = 42, *P* < 0.0001), and showed temporal and age-specific variations. By radiotracking 60 bats for 10 months, we obtained the first estimates of minimum monthly survival for this species as 0.81 (95% CI = 0.74 to 0.86), but this could be an underestimate due to possible undetected emigration of tagged bats.

## Teaser

Demography of the Gambian Epauletted Fruit Bat based on 3-year data collected in Ghana to describe population dynamics, birth rates, and survival estimates of this species. First estimates of minimum monthly survival for this species are provided but possibly affected by emigration of tagged bats.

Demographic parameters are key to understanding the dynamics of populations (Pryde et al. 2005; Skalski et al. 2005; O’Shea et al. 2011; Luis et al. 2013). Parameters such as survival, population sizes, and birth rates are major determinants of growth or decline and overall viability of animal populations (Schorcht et al. 2009). For demographic research to be successful, the population under study should be easily observable and monitored over time (Humphrey and Oli 2015). However, this is often very challenging in free-ranging animals, making demographic studies very difficult to conduct (Manning and Goldberg 2010). This has been a major limiting factor, resulting in the paucity of demographic information for many species.

Among mammals, bats in particular are very difficult to monitor and observe because of their cryptic nature, the difficulty in capturing and counting them, and their nocturnal behavior (O’Shea et al. 2003, 2004). Recent advancements in technology such as radiotelemetry, the development of sophisticated models, and flexible software (Lebreton et al. 1992; Cooch and White 2006) have facilitated the study of complex dynamics in wildlife populations. In particular, capture–mark–recapture (CMR) models have been instrumental in the study of population dynamics, especially of ecologically cryptic species (Burnham et al. 1987; Pollock et al. 1990; Lebreton et al. 1992).

Despite the availability of such methods and the long history of bat research, population estimates for the majority of bat species are lacking. Although some notable efforts have been made to improve this knowledge gap, most of these studies have focused on insectivorous bat species (e.g., Sendor and Simon 2003; Papadatou et al. 2009; Schorcht et al. 2009; O’Shea et al. 2010, 2011; Humphrey and Oli, 2015). Similar studies for fruit bat populations are largely lacking. For instance, the first robust estimates of demographic parameters, such as survival, for any species in the family Pteropodidae were provided in 2012 (Hayman et al. 2012).

Baseline data on population demographics are urgently required for more members of the Pteropodidae as they are becoming increasingly globally threatened. Currently, 72 out of 193 extant and assessed species are listed as threatened (Critically Endangered, Endangered, Vulnerable) on the IUCN Red List, while 14 species are data deficient (IUCN 2022). There is evidence of range-wide population declines in over 89 species of pteropodid fruit bats, while population trends for 54 species are unknown. Fruit bat species are also of interest to zoonotic disease and public health scientists because of their links to emerging zoonotic diseases (Calisher et al. 2006; Drexler et al. 2012; Brook and Dobson 2015). An understanding of the population dynamics of these species is important for informing adaptive conservation management decisions (Beissinger and Westphal 1998) and for elucidating zoonotic pathogen dynamics and persistence in bats (O’Shea et al. 2011).

The Gambian Epauletted Fruit Bat (*Epomophorus gambianus*) is a common species widely distributed throughout much of West Africa, extending into parts of Central Africa and Ethiopia (Boulay and Robbins 1989; Happold 2013). In this study, we provide the first estimates of demographic parameters—specifically local population size, survival, sex ratios, and birth rates for a population of *E. gambianus* in Ghana.

## Materials and methods

Ethical approval was obtained for this study from the Ethical Approval Committee of the Noguchi Memorial Institute for Medical Research, Ghana (CPN:002/13-14). Bat sampling and handling techniques followed Sikes et al. (2016).

### Study site

All data for this study were collected from Ghana, West Africa, from September 2012 to August 2016. We focused on a large *E. gambianus* colony at Ve-Golokuati (06°59.851ʹN, 000°26.218ʹE) in the Volta region of Ghana (hereafter VG) as part of previously ongoing studies (Amponsah-Mensah et al. 2019, 2022). Assessments of similar large roosts identified previously (Amponsah-Mensah 2017) also showed that this roost appears to be representative of the species. The large number of roosts within the colony also made it convenient to collect a large quantity of data on the species for our assessments.

### Bat trapping and sampling

Bats were caught using ground mist nets (3 to 5 m high, 6 to 18 m long) from November 2012 to October 2015. Trapping lasted from 1 to 5 nights per month between 18:00 and 06:00 h GMT each night for a total trapping effort of 5,358 net hours (1 net hour = 6 m net opened for 1 h). Each bat caught was held in a separate cloth bag and taken to a nearby sheltered area to be processed before release at the exact site of capture. Each bat caught was marked with either a thumb band (Tidemann 1999; Hayman et al. 2012) or a Trovan RFID nano transponder (Trovan Electronic Identification Systems, United Kingdom) implanted subdermally in the dorsum (Neubaum et al. 2005) using a pistol implanter before being released. The weight and forearm length of each bat were recorded. Sex was determined by using sex-dimorphic features and by carefully examining external genitalia using the shape of the genital opening (horizontal slit for females vs. round for males; Supplementary Data SD1). Reproductive status and age category were determined by visual examination of morphological features (Boulay and Robins 1989). All bats were categorized into juvenile, sexually immature adult, or adult.

### Population estimates

Colony size at the main study site was estimated monthly over 3 years (October 2012 to October 2015) by counting individual bats in each roost tree using a handheld trip counter. This was done by a single observer (KA-M) throughout the study to maintain consistency and increase internal validity. Counts were usually started after 08:00 h when bats had returned to roosts and movement at roosts was minimal, and were completed by 16:30 h before bats began to leave roosts to forage.

### Estimation of age-specific sex ratios

Assuming an equal chance of capturing individuals irrespective of age or sex, sex ratios (with 95% CI) were calculated for each of the 3 age classes: juveniles, sexually immature, and adults. Sex ratios were expressed as the ratio of the number of males to the number of females for a multiple-sampling event with replacement, using the methodology described by Skalski et al. (2005). Sex ratios were assessed to determine if there was variation from a 1:1 ratio among the different age classes and within the overall colony, using chi-square goodness-of-fit (GOF) tests.

### Estimation of birth rates, lactation, and weaning periods

*Epomophorus gambianus* is reported to breed twice a year with females normally giving birth to 1 young per reproductive season (Thomas and Marshall 1984; Boulay and Robbins 1989). To assess this in our study population, all females that were caught during each monthly trapping session were examined for pregnancy (by abdominal palpation) and lactation (by the expression of milk from the nipples). For each reproductive season, we selected the 2 consecutive months with the highest proportion of females in each reproductive state (pregnant, lactating) as the peak pregnancy and lactation periods and used these to estimate pregnancy and lactation rates. The pregnancy and lactation rates were used as a proxy to provide 2 separate estimates of birth rates for each reproductive season, based on the assumptions that all detected pregnancies resulted in the successful live birth of a single pup and that all females that were detected to be lactating had successfully given birth following pregnancy. Chi-square tests and Fisher’s exact test were used, where appropriate, to test for differences in pregnancy and lactation rates between seasons and years.

During monthly colony counts, lactating females could easily be seen at day roosts with their young attached to them and the number of such lactating females were also counted. This, together with monthly estimates of the proportions of females detected to be lactating or pregnant, was used to determine the birthing periods, lactation periods, and weaning periods for *E. gambianus.*

### CMR and estimation of survival rates

Passive integrated transponder (PIT) tags were initially used to mark bats, but these resulted in low recapture rates of less than 3% of over 2,300 tagged bats. Hence, radiotelemetry was used to mark and locate bats for CMR analyses. Sixty bats (14 adult males, 16 adult females, 28 sexually immature adult males, and 2 sexually immature adult females) were fitted with SOM 2190 HWSC radio transmitters (Wildlife Materials International, Inc.). Selection of bats for radio-tagging was based on weights of the bats to ensure that tag weights did not exceed recommended tag-to-bat weight ratio for telemetry (Amelon et al. 2009; O’Mara et al. 2014). Our tags weighed on average 5.1 ± 0.5% (range: 4.0% to 6.8%) of the total body weight of tagged bats. We fitted 20 tags in October 2015 and 40 tags in February 2016. Tagged bats were subsequently tracked during the day at least once a month over a period of 10 months using a TRX-1000S receiver and a 3-element directional Yagi antenna (Wildlife Materials International, Inc.). To ensure presence data were not erroneously collected from tags that had fallen off bats, tagged bats were tracked to their day roosts to visually confirm their presence within the colony.

Reencounter data were converted to encounter histories for each bat, representing whether a bat was encountered (1) or not encountered (0) during each radio-tracking session. Encounter histories were used for the estimation of apparent monthly survival and recapture probabilities. Apparent survival (Φ) is the probability that an animal survives from one sampling occasion to the next and remains available for recapture within the study population, while encounter probability (*p*) is the probability that a marked animal will be encountered over a specified time interval on condition that it is alive and within the study population (see Cooch and White 2006). CMR analyses are unable to distinguish between mortality and emigration from the study site; thus, all survival estimates are “apparent” rather than true. Radiotracking of bats was carried out between October 2015 and August 2016, but only data from the first 8 months were used in the analyses because, beyond this, the battery life of the first group of tags was too low to detect bats accurately.

CMR data were analyzed using the program Mark (White and Burnham 1999) by fitting the Cormack–Jolly–Seber (CJS) model to the data in R using “RMARK” (Laake 2013). The CJS model allows estimation of apparent survival and recapture probabilities in open populations provided that the data meet specific assumptions which can be tested using a GOF test (Lebreton et al. 1992). Each bat was categorized under 1 of 2 age classes (adult and sexually immature adult) and into 1 of 2 sex classes (male and female). Survival was modeled as a constant, and as a function of age and sex, both additively and interactively. Recapture rates were modeled as constant, a function of sex and age (both additively and interactively), and separately for each recapture occasion.

An initial GOF test was performed for the global model using the program “RELEASE” and found no indication of lack of fit (χ^2^_12_ = 10.77, *P* = 0.5), but showed slight under-dispersion of the data (TEST 1 + TEST 2/df = 0.89). A variance inflation factor (*ĉ*) of 1 was maintained for the analyses (Cooch and White 2006). Model selection was based on the Akaike information criterion adjusted for small samples (AICc). To account for model uncertainty, weighted model averaging for candidate models was used to provide robust parameter estimates and their unconditional 95% CI (Burnham and Anderson 2002) in the event where no particular model received overwhelming (>95%) Akaike weight.

## Results

### Monthly estimates of the size of the study colony

**Fig. 1.**
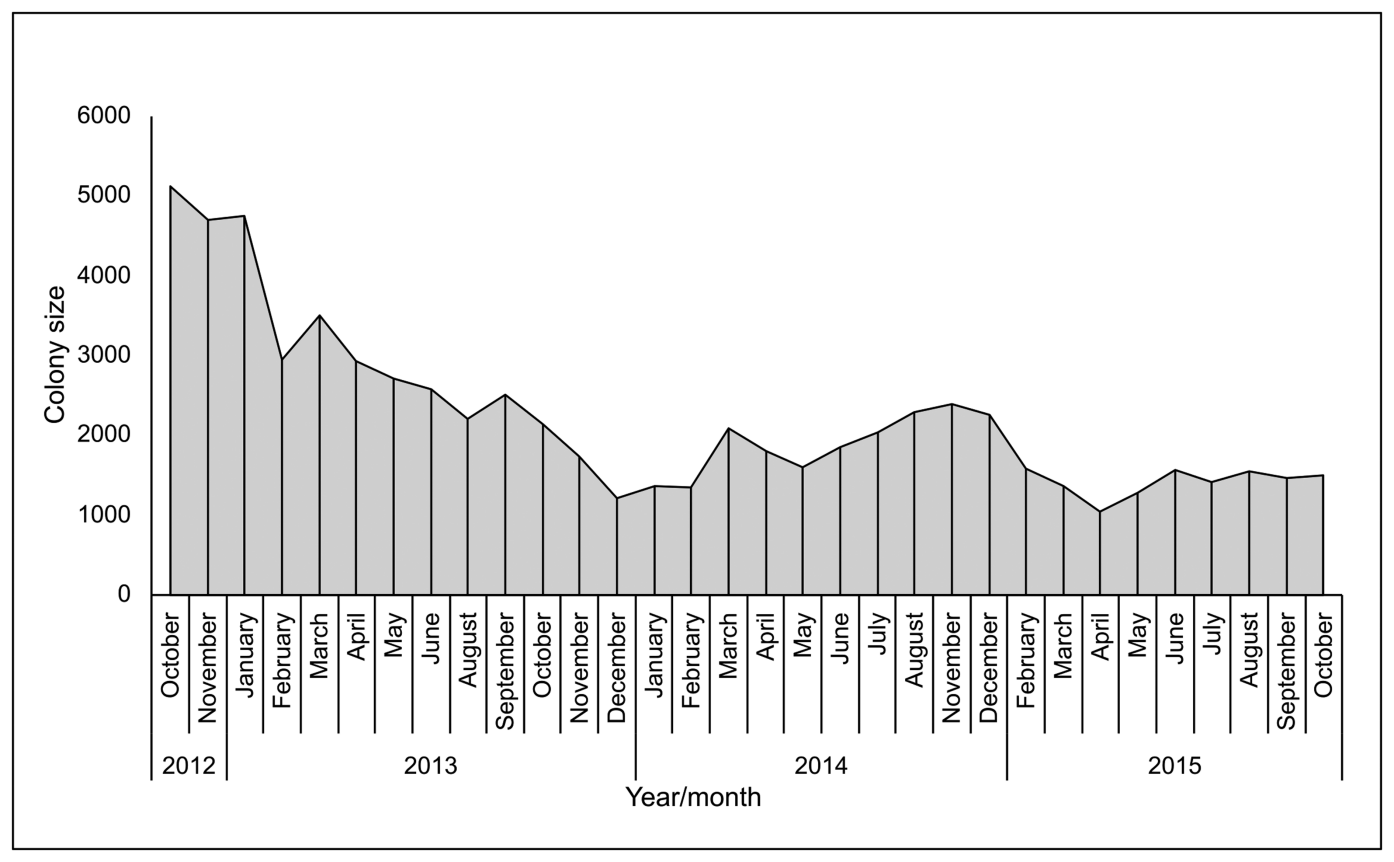
Monthly *Epomophorus gambianus* population estimates based on manual counts. No counts were conducted for December 2013, July 2013, September 2014, October 2014, and January 2015.

Monthly population size estimates of the VG colony varied between a minimum of about 1,000 to a maximum of 5,000 bats, with peak numbers occurring in March and September each year (Fig. 1).

### Sex ratios

Sex ratios for the VG colony were based on a total of 2,551 bats trapped between 2013 and 2015. Since trapping of bats occurred during nighttime feeding (and not at roost sites) and considering that there is no evidence of aggregation by demographic features during nighttime movements by this species, there was minimal chance of violating our assumption of an equal chance of capturing individuals of both sexes. It is also unlikely that the sexes of individuals were misidentified given our experience in assessing the sex of thousands of adults and juveniles of this species and other closely related species. The overall sex ratio for captured bats was significantly male-biased (χ^2^_1_ = 85, *P* < 0.0001), with a sex ratio of 1.45 (95% CI: 1.34 to 1.56; Table 1). There was no difference between the 3 years in the proportions of males and females captured (χ^2^_2_ = 2.4, *P* = 0.3). The proportions of males and females however differed significantly between the 3 age classes (χ^2^_2_ = 264, *P* < 0.0001) with sex ratios differing significantly from a 1:1 ratio for each age class: 58% of all juveniles were males (χ^2^_1_ = 26, *P* < 0.0001) while over 78% of the sexually immature adults were males (Table 1). The adult sex ratio, however, was significantly female-dominated, with 62% of all adults assessed being female (χ^2^_1_ = 42, *P* < 0.0001).

**Table 1.** Age-specific sex ratios (proportion of males to females) of the *Epomophorus gambianus* colony at Ve-Golokuati.

Age category	Number of individuals captured	Sex ratio M:F (95% CI)
Juvenile	980	1.39 (1.18 to 1.63)
Sexually immature adult	847	3.63 (2.81 to 4.69)
Adult	724	0.61 (0.46 to 0.82)
Total	2,551	1.45 (1.34 to 1.56)

**Fig. 2.**
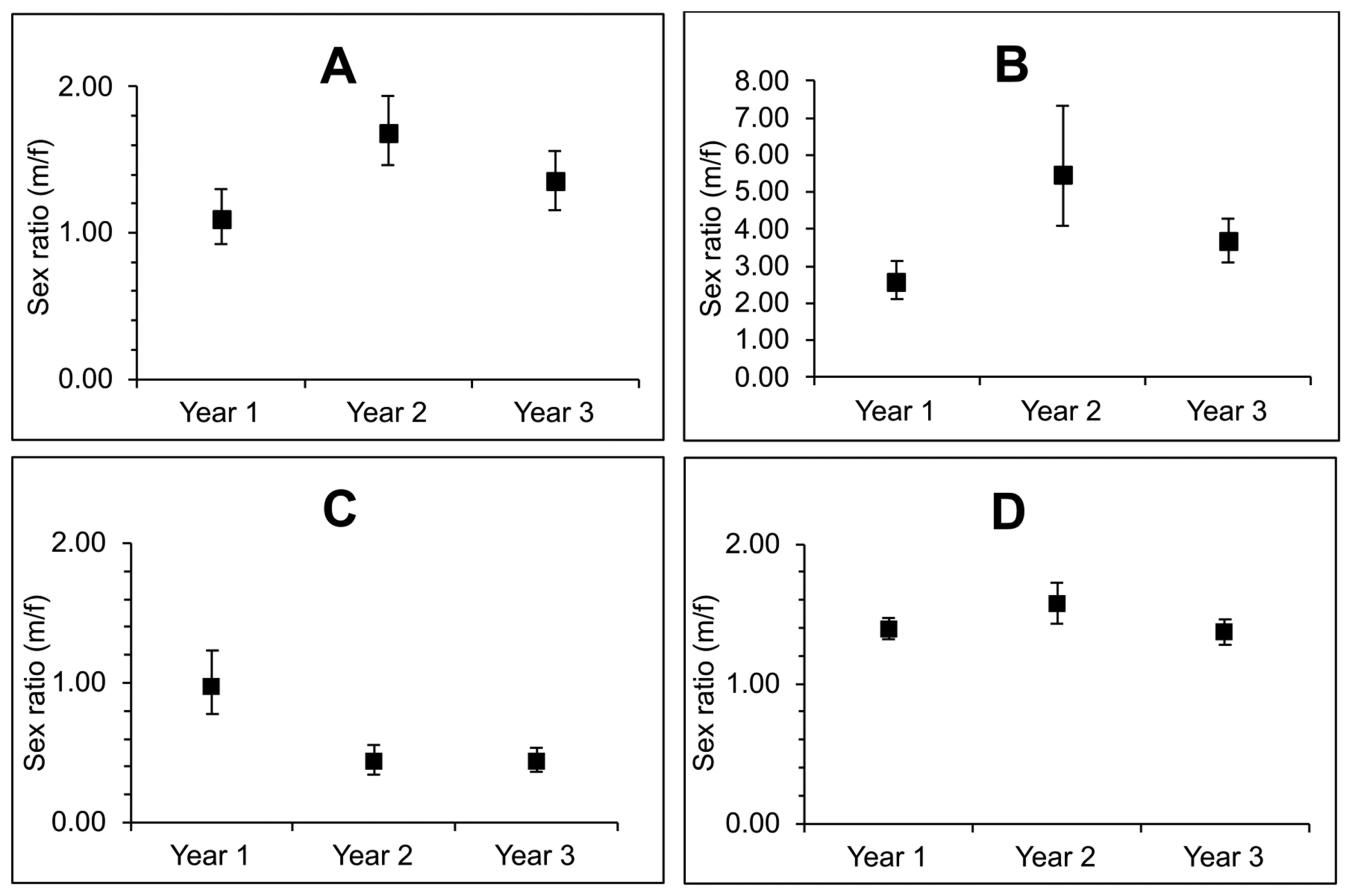
Annual variation in age-specific sex ratios for *Epomophorus gambianus*: (A) juveniles; (B) sexually immature adults; (C) adults; (D) total sex ratios. Vertical lines are 95% confidence intervals.

For each age class, significant variations in sex ratios for *E. gambianus* were detected between each of the 3 years of the study (juveniles, χ^2^_2_ = 7.3, *P* = 0.03; sexually immature adults, χ^2^_2_ = 13.7, *P* = 0.001; adults, χ^2^_2_ = 26, *P* < 0.0001). Similar to the overall sex ratio, both juvenile and sexually immature adult sex ratios were male-dominated in all 3 years, but the ratios varied by year (Fig. 2A and B). Adult sex ratio, however, varied from an approximately 1:1 ratio in the first year to a significantly female-biased sex ratio in the second and third years (Fig. 2C).

### Reproduction

**Fig. 3.**
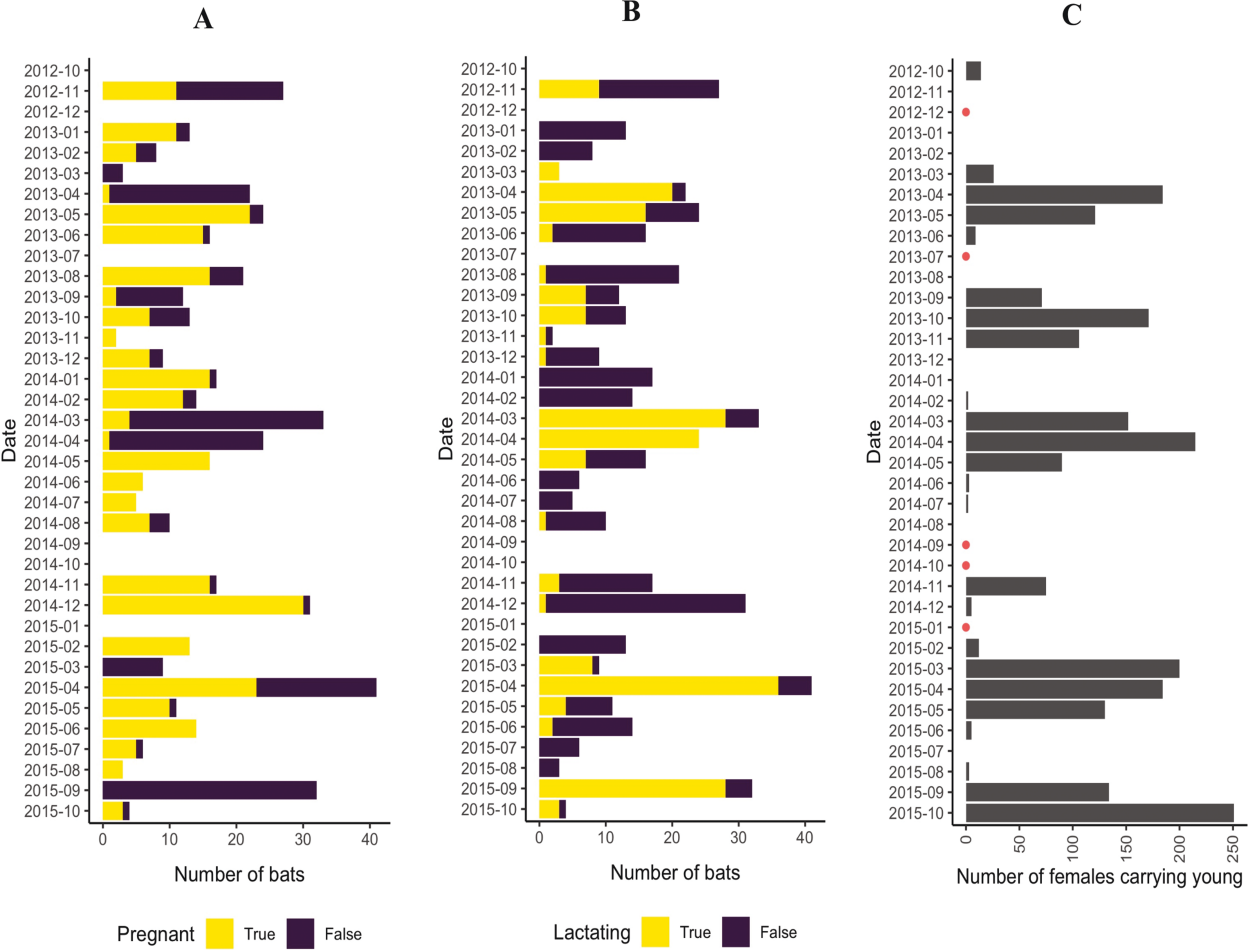
Seasonality in reproduction for *Epomorphous gambianus* females over 3 years (2012 to 2015) indicating: (A) monthly number of captured females detected to be lactating; (B) monthly number of captured females detected to be pregnant; (C) number of females observed to be carrying young at roosts during monthly roost counts. Circles indicate months where no counts were conducted.

Two reproductive seasons were observed every 12 months for *E. gambianus*, with births followed almost immediately by another embryonic development and pregnancy; a newly formed fetus could be detected in female bats roughly a month after birth periods. In each year, pregnancy in the first reproductive season commenced around April/May and births occurred approximately 4 months later (in August/September) when lactation was first detected in captured females (Fig. 3). Pregnancy for the second reproduction season occurred around October and lasted for about 5 months with births occurring in March/April when lactation was detected in captured females (Fig. 3). At this time, females were observed at roosts with newly born pups.

Lactation lasted for a period of 3 to 4 months for each reproductive season during which females could be observed carrying their young at day roosts. We detected that 75 lactating females were also in the early stages of pregnancy, which was around 2 months (May to June and October to November) before weaning. Weaning occurred in December and June each year. The proportion of females that were lactating correlated strongly and positively with monthly counts of the number of females that were observed to be carrying their offspring at roost sites (Fig. 3C); hence, the latter provided good support for estimates of lactation rates and periods.

### Birth rates

A total of 371 adult females were assessed during peak pregnancy and lactation periods for signs of pregnancy or lactation to estimate pregnancy and lactation rates. Pregnancy rates were estimated for 6 reproductive seasons while lactation rates were estimated for 5, as no sampling was done during the peak lactation period for the second reproductive season of the second year. During the second reproductive season of the first year, the proportion of lactating females was significantly lower compared to the proportion of females that were found to be pregnant in that same reproductive season (Fisher’s exact test, *P* = 0.001). Pregnancy and lactation rates for all the other reproductive seasons were not statistically different (Fisher’s exact tests: Y1S1, *P* = 0.6; Y2S1, *P* = 1.0; Y3S1, *P* = 0.1; Y3S2, *P* = 0.4). The birth rate estimated using pregnancy rate as a proxy was 0.93 (95% CI = 0.88 to 09.6) young per reproductive season, while the birth rate estimated from lactation rate was 0.85 (95% CI = 0.79 to 0.90) per reproductive season across the 3 years (Table 2).

**Table 2.** Seasonal proportions of total adult female *Epomophorus gambianus* sampled that were observed to be pregnant and lactating. Figures in parentheses indicate the total number of females sampled during the peak pregnancy/lactation period.

Reproductive status	Pregnancy		Lactation	
Year/reproductive season	Season 1	Season 2	Season 1	Season 2
Year 1	0.76 (21)	0.93 (40)	0.92 (25)	0.56 (25)
Year 2	0.90 (31)	1.00 (22)	0.91 (57)	—
Year 3	0.98 (44)	0.95 (20)	0.88 (50)	0.86 (36)
Total (per season per year)	0.93 (95% CI: 0.88 to 0.96)		0.85 (95% CI: 0.79 to 0.90)	

### Radiotracking and CMR analyses

From CMR analyses, no model received overwhelming support (top model Akaike weight 0.19), so model averaging was used to generate optimal parameter estimates given model uncertainty. There was moderate support for both sex and age class differences in survival probability (summed Akaike weight = 0.76). All of the best models contained constant estimates for recapture probability through time (Table 3) and so only a single estimate was provided for each group.

**Table 3.** Summary of CJS capture–recapture models for *Epomophorus gambianus* showing the best models (summed model weight > 0.95). φ = apparent survival; *p* = encounter probability. Models are ranked by ascending AICc values. AICc is the Akaike information criterion corrected for small samples, *k* is the number of parameters, *w* is the Akaike weights of the models, and ΔAICc is the difference between the AIC of each model and that of the most parsimonious model. “+” indicates additive effects, “*” indicates interactive effects, and “.” indicates constant.

Model	AICc	ΔAICc	*w*	Deviance	*k*
φ(Age class * Sex)*p*(.)	269.051	0.000	0.190	88.022	5
φ(Age class + Sex)*p*(.)	269.430	0.379	0.157	90.534	4
φ(Age class * Sex)*p*(Sex)	270.535	1.483	0.090	87.344	6
φ(Age class + Sex)*p*(Sex)	270.831	1.779	0.078	89.802	5
φ(Age class)*p*(.)	271.086	2.035	0.069	94.296	3
φ(Age class * Sex)*p*(Age class)	271.161	2.109	0.066	87.970	6
φ(Age class + Sex)*p*(Age class)	271.486	2.435	0.056	90.457	5
φ(Age class)*p*(Age class * Sex)	272.257	3.206	0.038	89.067	6
φ(Age class + Sex)*p*(Age class * Sex)	272.282	3.230	0.038	86.901	7
φ(Age class * Sex)*p*(Age class + Sex)	272.282	3.230	0.038	86.901	7
φ(Age class + Sex)*p*(Age class + Sex)	272.316	3.265	0.037	89.125	6
φ(Age class)*p*(Sex)	273.038	3.987	0.026	94.142	4
φ(Age class)*p*(Age class)	273.166	4.114	0.024	94.270	4
φ(.)*p*(Age class * Sex)	273.688	4.637	0.019	92.659	5
φ(.)*p*(.)	273.996	4.944	0.016	99.284	2
φ(Age class * Sex)*p*(Age class * Sex)	274.501	5.450	0.012	86.901	8

The model-averaged parameter estimates of monthly survival for adult males was 0.91 (95% CI = 0.75 to 0.97); for adult females was 0.82 (95% CI = 0.67 to 0.91); and for sexually immature adult males was 0.74 (95% CI = 0.62 to 0.84). Due to the very low number of sexually immature adult females that could meet the weight requirements for radio-tagging (*n* = 2), the parameter estimates for this group are unreliable and hence omitted. The recapture probability was very similar among groups (Supplementary Data SD2).

## Discussion

In this study, we monitored a colony of *E. gambianus* over 3 years and provide the first estimates of demographic parameters for a colony of *E. gambianus* and one of a few studies that provide such estimates for a fruit bat (Pteropodidae). This highlights the general lack of information and paucity of estimates of demographic parameters for fruit bats, especially considering that many species are threatened or undergoing population declines.

### Colony size and population dynamics

It is uncertain what caused the decrease in population size after the first year of monitoring. No excessive mortality was observed during the study period that could explain such a large drop in colony population size. Although hunting of fruit bats is common, particularly in southern Ghana (Kamins et al. 2011; Leach et al. 2017; Ohemeng et al. 2017), only a very low level of hunting involving isolated incidents of the shooting of individual bats by children with catapults was observed at the VG colony, which alone is unlikely to have caused the observed decrease in numbers. During the period of the 2014 Ebola outbreak in West Africa, we did observe that some roost trees in the study area were cut down or trimmed frequently due to heightened fear of bats. This resulted in the permanent loss of some of the roosting trees and the displacement of bats which could have also contributed to the population decline.

It is also likely that disturbance caused by monthly trapping from this colony during this study could potentially lead to the relocation of bats to other colonies over the study period. The combined effect of disturbance at roosts and from this study, and the loss of roost trees are likely to have caused the observed reduction in the population at this colony by increasing mortality and/or the emigration of bats from this colony. Declines in other bat populations for similar causes have been reported (Mickleburgh et al. 1992, 2002).

### Variation in sex ratios

Sex ratio is an important demographic parameter that is key to understanding the health and persistence of populations (Skalski et al. 2005) but this parameter is mostly unavailable for most African pteropodids. Our study shows that while the overall sex ratio for all captured bats was significantly male-dominated, annual variations in sex- and age-specific ratios were observed. In several mammalian species, sex ratios vary from unity (Barclay 2012) and in bats it is often skewed toward males (Perry et al. 2010). A common explanation for skewed sex ratios is based on adaptive theories. Arguments by Trivers and Willard (1973) predict that in polygynous species, mothers able to provide greater than average resources should invest more in offspring of the sex that benefits most from the added investment; essentially, mothers in good condition or with abundant resources should produce male, whereas parents in inferior condition or limited resources should produce female offspring. This hypothesis is supported to varying degrees by different studies. Some authors argue that certain mechanisms that influence sex ratios may not be adaptive, while others demonstrate that predicting birth sex ratios can be a complex interaction of several factors. For instance, Barclay (2012) found that the juvenile sex ratio in the Big Brown Bat (*Eptesicus fuscus*) showed complex seasonal and annual variations. This variation depended on environmental and maternal factors and was also likely to differ geographically due to varying lengths of the season. Barclay (2012) emphasized that such complexities can only be fully understood through long-term studies, and therefore we cannot assign similar explanations to our observed sex ratios without such long-term data.

Bimaturism in *E. gambianus* is a plausible justification for the male-biased sex ratio in sexually immature adults. Bimaturism has been reported in some fruit bat species including the Hammer-headed Bat (*Hypsignathus monstrosus*; Bradbury 1977), the Grey-headed Flying Fox (*Pteropus poliocephalus*; Welbergen 2010), Peter’s Dwarf Epauletted Fruit Bat (*E. pusillus*), and Buettikofer’s Epauletted Fruit Bat (*Epomops buettikoferi*; Thomas and Marshall 1984). For *E. buettikoferi*, a species of similar size to *E. gambianus*, Thomas and Marshall (1984), observed that females became sexually mature at 6 months, while males only reached puberty at 11 months. Recapture data from the current study suggest that bimaturism occurs in *E. gambianus* as well, with males maturing into adults when they are about 12 months old, while females can become pregnant at about 5 months of age (Supplementary Data SD3). Because the age at sexual maturity in males seems to be almost twice that of females, it is expected that more males will be in this sexually immature adult “transition period” compared to females, even if the initial sex ratio at birth was 1:1. Hence, the highly male-skewed sex ratio in the sexually immature adult age class in *E. gambianus* likely is a result of delay in male maturation, in addition to an already male-biased juvenile sex ratio.

Temporal variations in sex ratios may, however, arise due to differential emigration or immigration of the sexes (Lovich and Gibbons 1990) or as a result of spatiotemporal differences in patterns of abundance in sexes (Perry et al. 2010). We propose that the substantial change in the adult sex ratio observed during the second year of this study could likely be a result of the emigration of adult males from the study area which is in part supported by a lower estimate of recapture probabilities for adult males than adult females in our CMR analyses and part by the lower estimate of the population size of the colony. In polygynous bats such as *E. gambianus*, males may emigrate to other colonies in an attempt to find suitable females, thus causing possible temporal variations in colony-specific sex ratios.

### Birth rates and reproductive chronology

Our findings confirm the reported reproductive chronology of *E. gambianus* as continuous bimodal polyestry with postpartum estrus (Thomas and Marshall 1984; Boulay and Robbins 1989) with an estimated birth rate of 0.89 (95% CI = 0.85 to 0.92) per female per reproductive season. We observed the gestation period to last 5 to 6 months, with a relatively shorter lactation period of 3 to 4 months (9 to 16 weeks)—both of these correspond to those reported in other studies (Thomas and Marshall 1984; Nowak 1999; Happold 2013). We observed births occurring in March/April and August/September each year, coinciding with the onset of the 2 rainy seasons for the study area. Yeboah (2008) reported breeding periods of *E. gambianus* as July/August and March/April in the Abirem District of the Eastern Region in Ghana. Marshall and McWilliam (1982) suggested that births of *E. gambianus* in Mole National Park occurred at the beginning of the rains in April. Elsewhere in West Africa, *E. gambianus* births have been reported to occur in April to May and October to November (Boulay and Robbins 1989; Nowak 1999; Happold 2013). Although most reports available on the birth periods of *E. gambianus* in West Africa were inconclusive, the majority affirms birth pulses that are synchronized with the bimodal rainy seasons, which correspond with food abundance. The synchronization of parturition with food abundance (and rainfall) is commonly reported and well-illustrated among the Pteropodidae in Africa (Happold and Happold 1990; Cumming and Bernard 1997).

Interestingly, our findings show a relatively longer (5-month) pregnancy period for the second reproductive season compared to the first period that lasted 4 months. Birthing in bats has been proposed to be timed such that food is available for juveniles after weaning (Fleming et al. 1972; Cumming and Bernard 1997). In a previous publication, Amponsah-Mensah et al. (2019) have shown peaks in the abundance of flowers and fruits occurring in June/July and December/January within the current study location; both periods coinciding with the weaning periods as reported in this study. We propose the possibility that the second pregnancy period is longer to ensure that the postweaning period coincides with peaks in food abundance. This current study, however, cannot explicitly explain the underlying mechanisms for this and further research is required to adequately test this hypothesis.

### CMR and survival estimates

Survival rate is an important demographic parameter that is useful in predicting the health of populations and also in assessing how populations fare under different stress factors. In free-ranging animal populations, particularly in bats, estimation of this parameter can be difficult because marking methods such as banding or PIT tags (as used in the initial stages of this study) require recapturing marked individuals but often result in low recapture rates (Towner et al. 2009), preventing robust estimation of survival and recapture parameters. The use of radio-tags appears to provide a solution to this problem, particularly in large tree-roosting fruit bats that are big enough to carry radio-tags with larger and longer-lasting batteries (several months). Thus, adhering to best practices concerning maximum tag weights for bats (O’Mara et al. 2014; Sikes et al. 2016) can preclude long-term tracking, especially in smaller species. Recapture rates from radio-tagging CMR analyses compared to initial recapture estimates from PIT tags (<3% of over 2,000 marked individuals) highlight the challenge associated with using tagging methods that require physical recapture of bats for CMR analyses.

Our CMR analyses provide moderate support for sex and age differences in survival rates, with males having a higher survival probability than females of similar ages. Survival rates often differ between sexes and age classes in free-ranging animals (Lebreton et al. 1992; Austad and Fischer 2016) with juvenile survival usually lower but increasing toward a stable survival rate at adulthood (Loery et al. 1987). Survival probabilities in bats are often lower in adult females, possibly due to extra energetic constraints associated with pregnancy and lactation (Kurta and Matson 1980; Perry et al. 2010). Also, the relatively larger size of adult *E. gambianus* males, a sexually dimorphic trait that occurs in the species (Boulay and Robbins 1989), may confer increased protection against predator attacks compared to females.

We estimated a monthly survival rate of 0.81 (95% CI = 0.74 to 0.86) for *E. gambianus* across all ages and sexes. However, our survival estimates for *E. gambianus* in this study could have been substantially underestimated as possible permanent emigration of some tagged bats were not distinguishable from death. Thirty-eight out of the 60 radio-tagged bats were not detected in the colony within 12 h after their initial release. Because tags were confirmed to be working before their release, it is unlikely that these tags failed immediately after release. Rather, we suspect that either these bats had emigrated from the study site as a result of the trauma of capture and tagging or were bats that did not belong to this colony but were only trapped in the study area during nomadic foraging trips. The VG colony likely forms part of a much larger network of *E. gambianus* colonies reported to occur across Ghana and individuals may mix freely among colonies (Riesle-Sbarbaro et al. 2018). Although long-distance nomadic movements are common among members of the family *Pteropodidae* (Tidemann and Nelson 2004; Richter and Cumming 2008; Breed et al. 2010; Roberts et al. 2012) and could have accounted for the low detection rates, we cannot rule out the potential effect of capture and handling trauma on attrition rate. Treating these individuals as transients and excluding them from the CMR analyses resulted in unreliable estimates with very wide confidence intervals as a result of the reduction in sample size (see Supplementary Data SD4). Our efforts to locate these bats up to distances of about 20 km from the study colony were unsuccessful. We propose that future studies should consider colony connectivity, with extra efforts to search and track tagged bats over much larger areas.

Monthly adult survival for *E. helvum* in Ghana, the only other African pteropodid species for which survival estimates are available (Hayman et al. 2012), was estimated as 0.96 (95% CI = 0.89 to 0.99). While it is very likely that emigration, rather than deaths, led to an underestimation of survival for *E. gambianus* in this study, we expect differences in life history traits (e.g., reproduction rates) to account for some differences in survival estimates between the 2 species. For instance, Lentini et al. (2015) observed that bats that produce fewer young each year had higher survival rates because of the energetic costs involved in pregnancy. On this basis, we would expect *E. gambianus* to have a lower survival rate because of its polyestrus reproduction compared to *E. helvum* which reproduces once a year.

Our study confirms the reproductive strategy of *E. gambianus* to be a bimodal polyestrus seasonal breeder with birth rates estimated at 0.89 (95% CI = 0.85 to 0.92) young per female per reproductive season. While the sex ratio of our study colony was largely male-biased, temporal variations and age-specific variations occurred with the latter possibly being influenced by bimaturism in this species. We estimate the monthly survival of *E. gambianus* across all ages and sexes to be 0.81 (95% CI = 0.74 to 0.86); the true estimates, however, are likely to be higher due to emigration from the study colony.

Estimates of demographic parameters are vital to understanding and predicting the viability of populations under different environmental factors, and for defining population trends, which currently remain unknown for *E. gambianus* (Tanshi and Fahr 2016). Considering the putative zoonotic importance of African fruit bats, these estimates are also essential to future analyses of infection dynamics in *E. gambianus*.

## Supplementary data

Supplementary data are available at *Journal of Mammalogy* online.

**Supplementary Data SD1.** Sexual dimorphic characteristics and external genitalia features of *Epomophorus gambianus* used in differentiating different ages and sexes. (A) *Epomophorus gambianus* adult female showing mammary gland. (B) External genitalia of adult female *E. gambianus*. Notice the leaf-like flap of the vulva with a slit-like orifice which upon careful and gentle flipping reveals the urogenital opening beneath. (C) *Epomophorus gambianus* juvenile female. (D) External genitalia of *E. gambianus* juvenile female. (E) *Epomophorus gambianus* adult male showing shoulder epaulette. (F) External genitalia of adult male *E. gambianus*. (G) *Epomophorus gambianus* juvenile male. (H) External genitalia of juvenile male *E. gambianus*. Notice the small round opening at the tip of the penis. (I) A sexually immature *E. gambianus* adult male showing a developing shoulder epaulette. (J) External genitalia of a sexually immature *E. gambianus* adult male.

**Supplementary Data SD2.** Model-averaged estimates for monthly recapture probability from the CJS model. Vertical bars are 95% confidence intervals.

**Supplementary Data SD3.** Selected capture data of bats recaptured at different ages after initially being caught as juveniles indicating different maturity periods for male and female *Epomophorus gambianus*. Proposed birth dates are arbitrary dates based on birth months in which births were observed for this study.

**Supplementary Data SD4.** Apparent survival estimates with 95% CI for radio-tracked bats after excluding transient bats from CMR analyses.

gyae096_suppl_Supplementary_Data_SD1

gyae096_suppl_Supplementary_Data_SD2

gyae096_suppl_Supplementary_Data_SD3

gyae096_suppl_Supplementary_Data_SD4

## Data Availability

The data that support the findings of this study are available from the corresponding author upon reasonable request.
