# Supplementary figures and images for: Demography of the Gambian Epauletted Fruit Bat (Epomophorus gambianus) in Ghana
Source: J Mammal. 2024 Sep 5;106(1):168–77. doi: 10.1093/jmammal/gyae096 (PMC11776427; doi:10.1093/jmammal/gyae096)

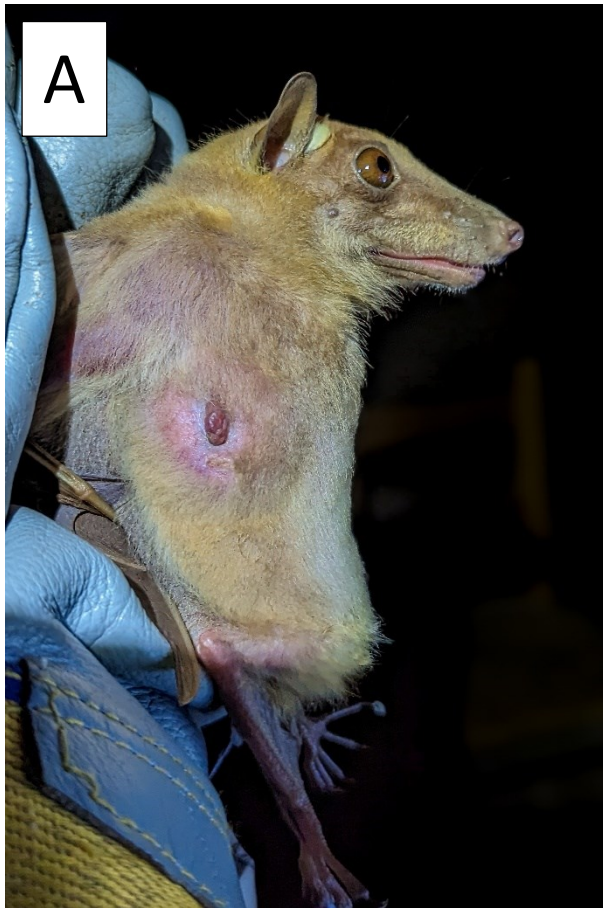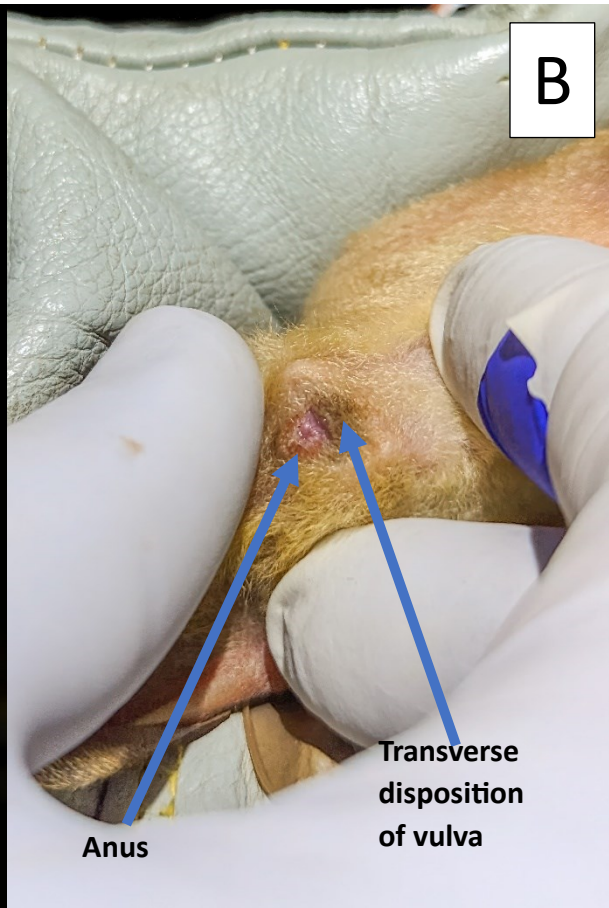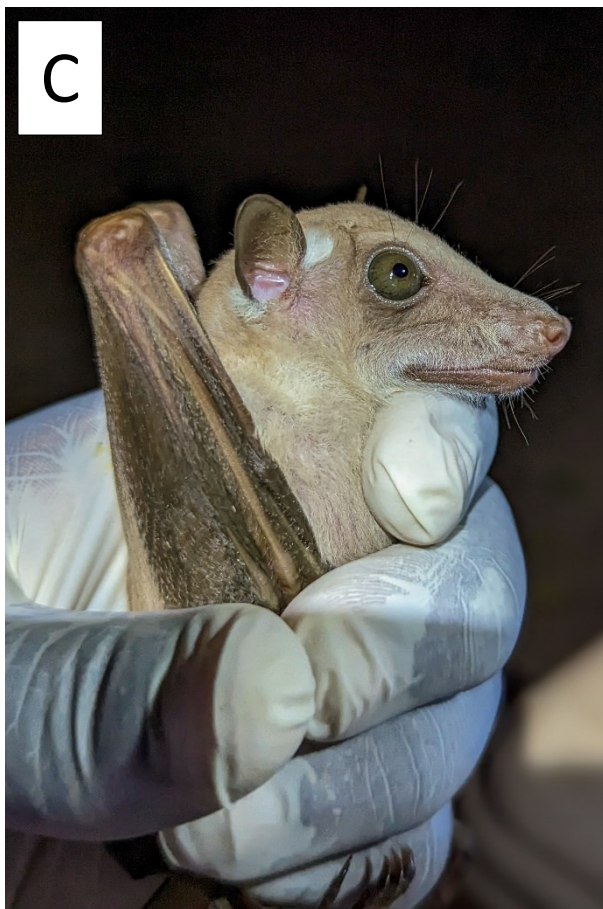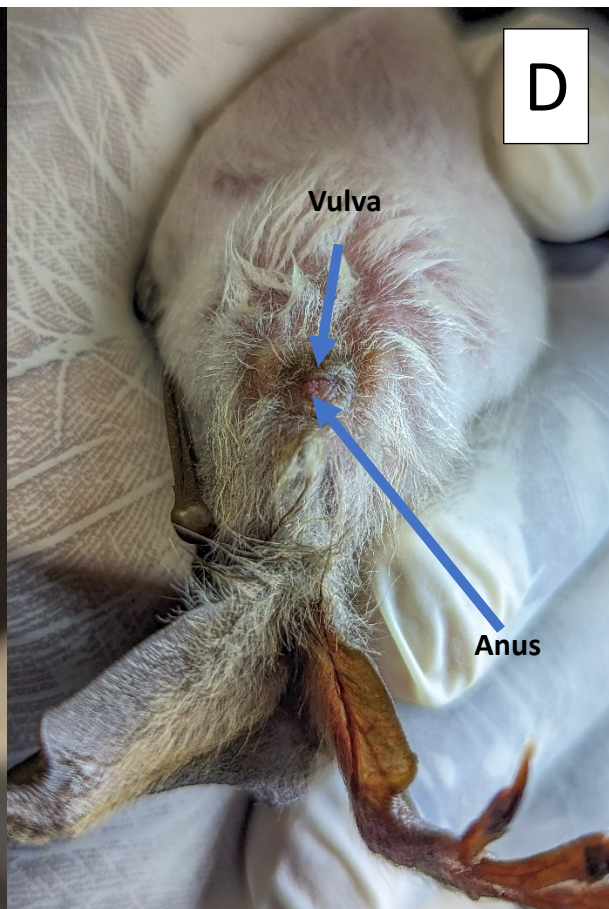

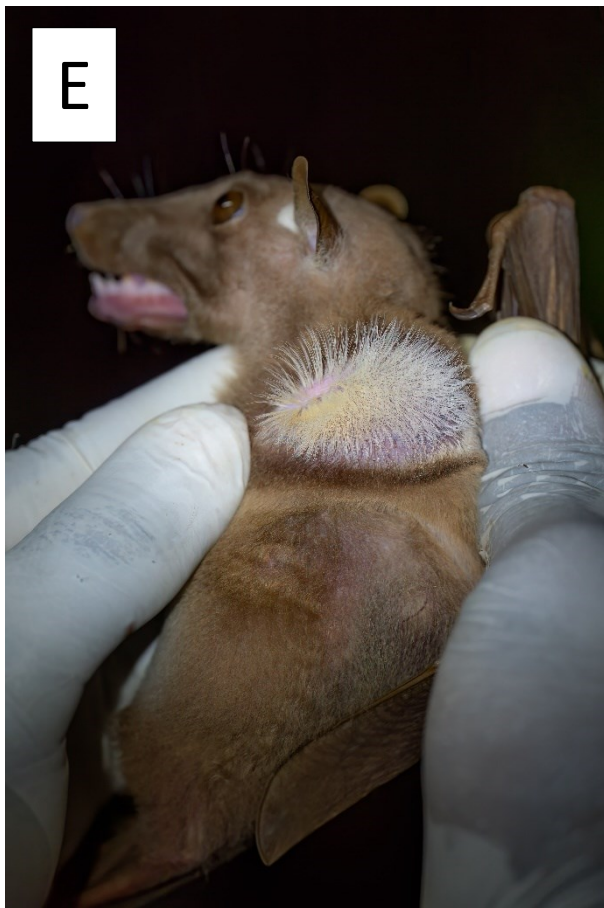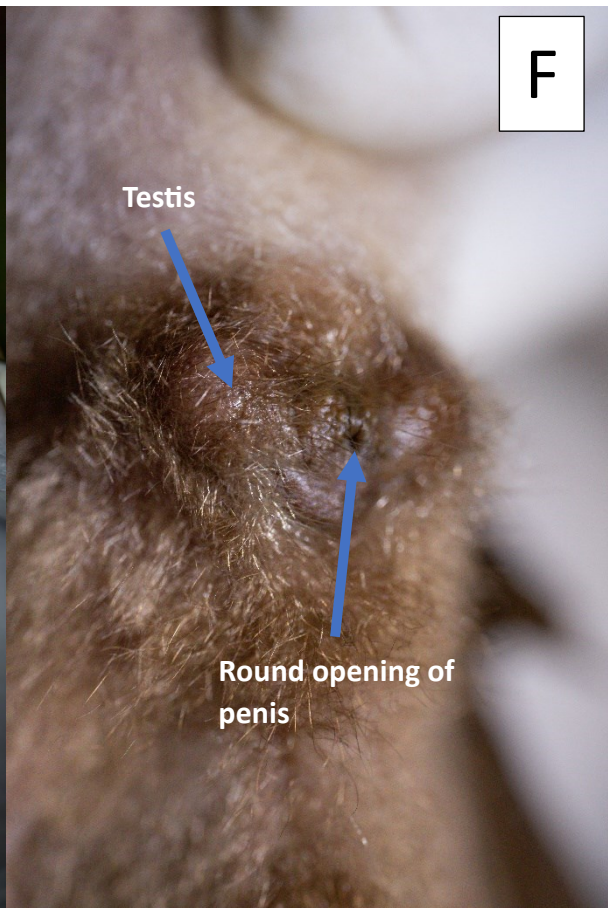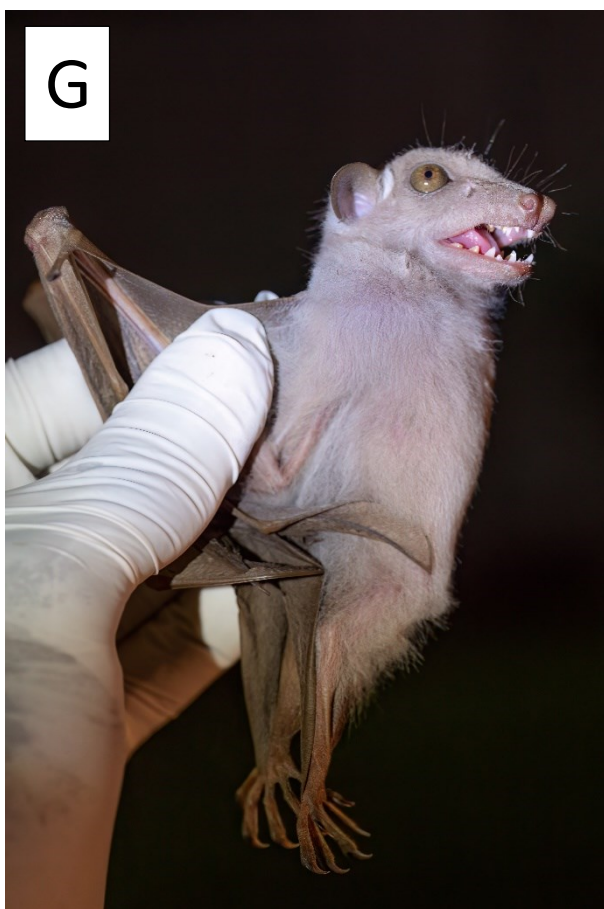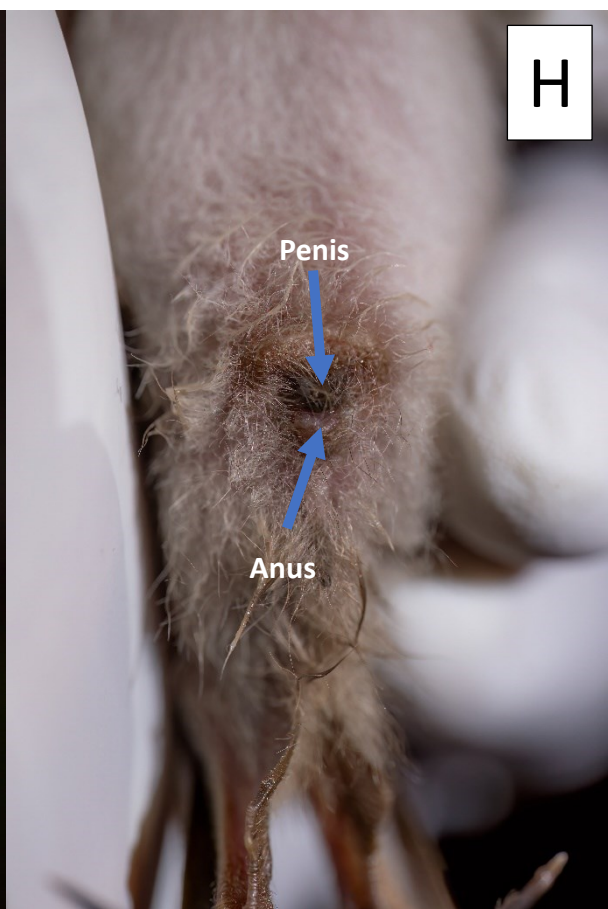

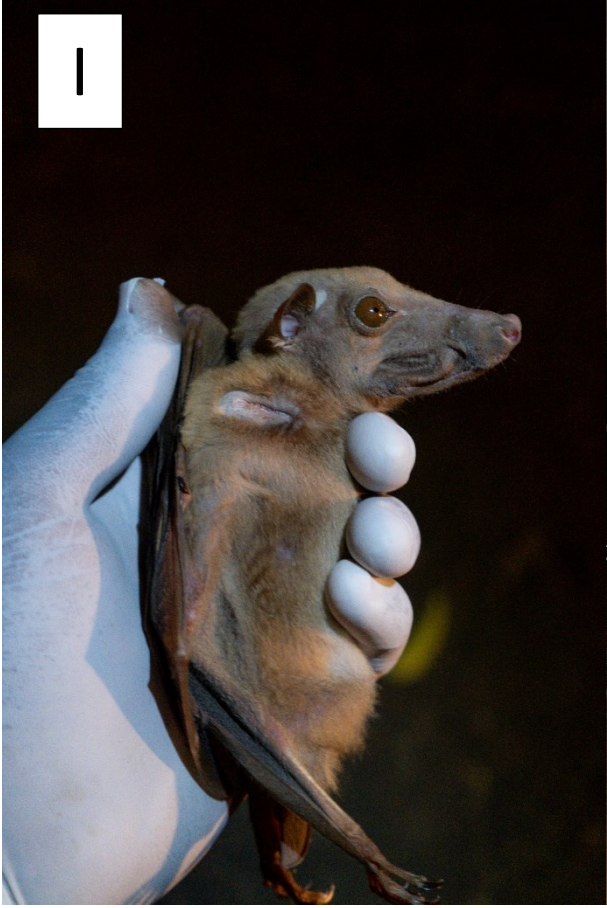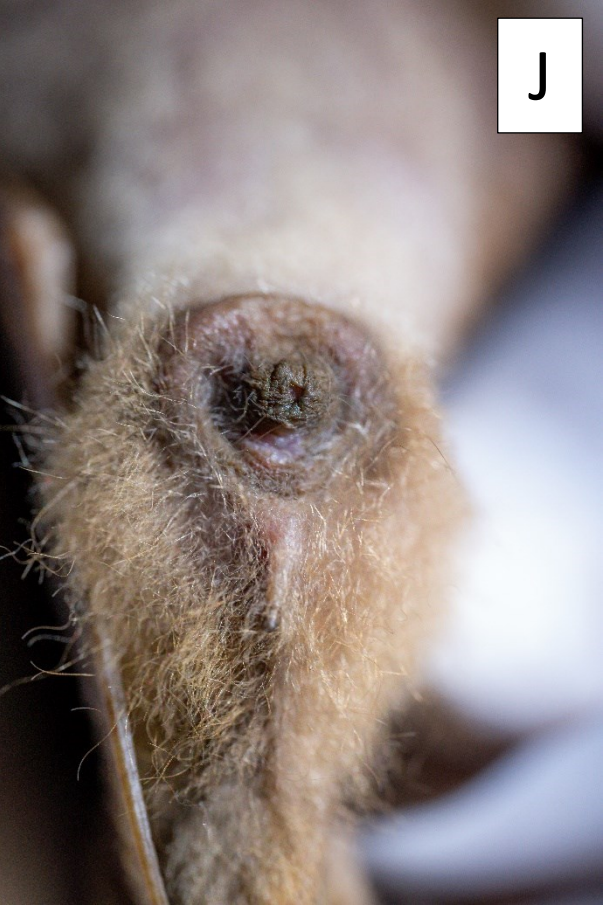

Supplement: gyae096_suppl_Supplementary_Data_SD1 [file gyae096_suppl_supplementary_data_sd1.pdf]

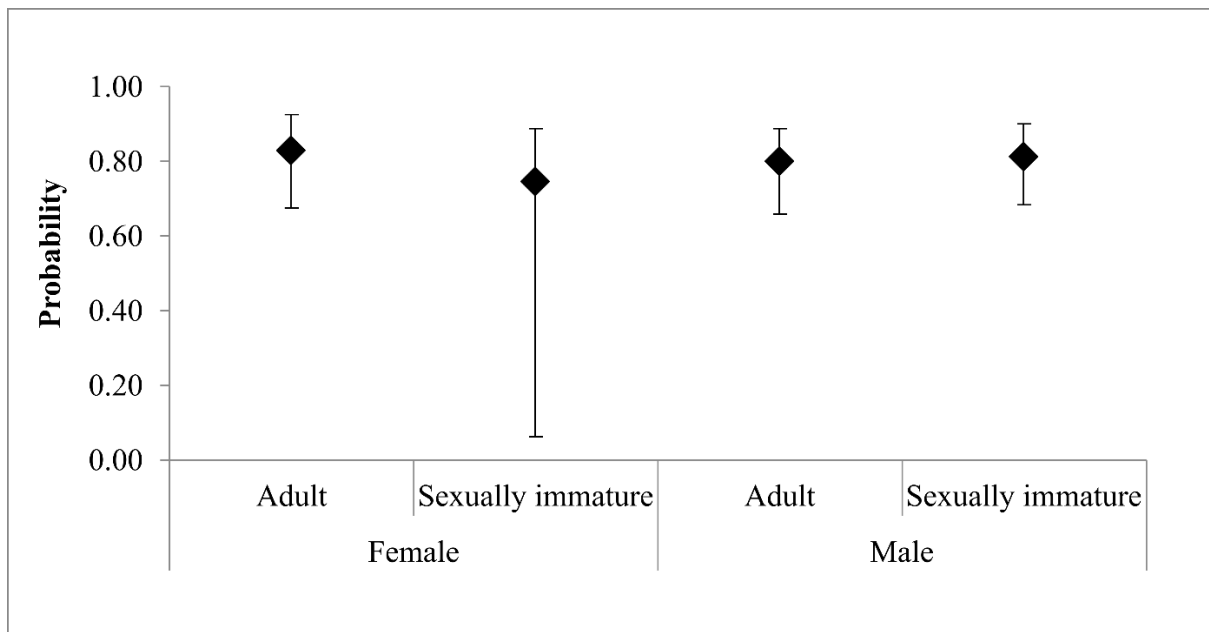

Supplement: gyae096_suppl_Supplementary_Data_SD2 [file gyae096_suppl_supplementary_data_sd2.pdf]
