# Supplementary material for: Demography of the Gambian Epauletted Fruit Bat (Epomophorus gambianus) in Ghana
Source: J Mammal. 2024 Sep 5;106(1):168–77. doi: 10.1093/jmammal/gyae096 (PMC11776427; doi:10.1093/jmammal/gyae096)
Supplement: gyae096_suppl_Supplementary_Data_SD3 [file gyae096_suppl_supplementary_data_sd3.pdf]

		First capture						Second capture						
**ID**	**Gender**	**Capture date**	**Age category**	**Forearm (mm)**	**Weight (g)**	**Estimated birth date**	**Estimated age (months)**	**Date**	**Age**	**Forearm (mm)**	**Weight (g)**	**Reproductive status at the second capture**	**The duration between captures (months)**	**Estimated age at second capture (months)**
N05742	F	21/05/2013	Juvenile	76	60	Mar-13	2	28/08/2013	Subadult	79.2	76		3	5
3456807	F	27/09/2013	Juvenile	74.5	66	Aug-13	1	19/02/2014	Adult	79.6	100	Pregnant	4	5
3585848	F	23/01/2014	Juvenile	73.2	64	Aug-13	5	29/04/2014	Adult	80.7	88		3	8
4434671	F	02/12/2014	Juvenile	72	54	Aug-14	4	30/03/2015	Adult		76		3	7
3479979	F	28/04/2014	Juvenile	71.2	46	Mar-14	1	27/04/2015	Adult	82.9		Lactating	11	12
A03382	F	30/10/2013	Juvenile	70.6	48	Aug-13	2	29/07/2014	Adult	86.6	126	Pregnant	8	10
A03382	M	30/10/2013	Juvenile	68.2	58	Aug-13	2	31/05/2014	Subadult	85.7	100		7	9
3491240	M	30/10/2013	Juvenile	70.6	51	Aug-13	2	29/08/2014	Subadult	81.7	92		9	11
3580986	M	29/04/2014	Juvenile	70.6	52	Mar-14	1	03/12/2014	Subadult	80.6	84		7	8
3586774	M	03/05/2014	Juvenile	76	62	Mar-14	2	21/12/2014	Subadult	85.6	104		7	9
3592286	M	31/05/2014	Juvenile	73	50	Mar-14	2	28/04/2015	Adult	90.5			10	12
4430652	M	03/12/2014	Juvenile	72	60	Aug-14	4	28/04/2015	Subadult	80.4			4	8
3585629	M	04/07/2014	Juvenile	74.3	64	Mar-14	4	02/12/2014	Subadult	80.7	88		4	8
