# Supplementary material for: Demography of the Gambian Epauletted Fruit Bat (Epomophorus gambianus) in Ghana
Source: J Mammal. 2024 Sep 5;106(1):168–77. doi: 10.1093/jmammal/gyae096 (PMC11776427; doi:10.1093/jmammal/gyae096)
Supplement: gyae096_suppl_Supplementary_Data_SD4 [file gyae096_suppl_supplementary_data_sd4.pdf]

		95% *CI*	
**Sex-Age class**	**Φ**	**Lower *CI***	**Upper *CI***
Female Adults	0.99	0.22	0.99
Male Adults	0.98	0.47	0.99
Male Juveniles	0.89	0.75	0.95
